# Microbial Synthesis and Transformation of Inorganic and Organic Chlorine Compounds

**DOI:** 10.3389/fmicb.2018.03079

**Published:** 2018-12-12

**Authors:** Siavash Atashgahi, Martin G. Liebensteiner, Dick B. Janssen, Hauke Smidt, Alfons J. M. Stams, Detmer Sipkema

**Affiliations:** ^1^Laboratory of Microbiology, Wageningen University & Research, Wageningen, Netherlands; ^2^Department of Biochemistry, Groningen Biomolecular Sciences and Biotechnology Institute, University of Groningen, Groningen, Netherlands; ^3^Centre of Biological Engineering, University of Minho, Braga, Portugal

**Keywords:** chlorine cycle, organochlorines, chlorine oxyanions, chlorination, dechlorination

## Abstract

Organic and inorganic chlorine compounds are formed by a broad range of natural geochemical, photochemical and biological processes. In addition, chlorine compounds are produced in large quantities for industrial, agricultural and pharmaceutical purposes, which has led to widespread environmental pollution. Abiotic transformations and microbial metabolism of inorganic and organic chlorine compounds combined with human activities constitute the chlorine cycle on Earth. Naturally occurring organochlorines compounds are synthesized and transformed by diverse groups of (micro)organisms in the presence or absence of oxygen. In turn, anthropogenic chlorine contaminants may be degraded under natural or stimulated conditions. Here, we review phylogeny, biochemistry and ecology of microorganisms mediating chlorination and dechlorination processes. In addition, the co-occurrence and potential interdependency of catabolic and anabolic transformations of natural and synthetic chlorine compounds are discussed for selected microorganisms and particular ecosystems.

## Introduction

Chlorine is the 20th most abundant element in the Earth’s crust (Winterton, 2000). The main form of chlorine on Earth is in sodium, potassium, and magnesium minerals such as halite, sylvite and carnallite, and as dissolved chloride in the oceans. Elemental chlorine (Cl_2_), which is industrially produced from brine by electrolysis, has become an essential reagent in chemical industries for the production of organochlorine compounds such as chlorine herbicides (e.g., 2,4-dichlorophenoxyacetic acid, 2,4-D), antimicrobial agents (e.g., triclosan), plastics [e.g., polyvinyl chloride (PVC)] and degreasing agents (e.g., chlorinated ethenes). Similarly, inorganic chlorine compounds have been widely used in the electronic, chemical and paper industries, either as part of the industrial product itself or for synthesis of intermediates for non-chlorine products (Fauvarque, 1996). Chlorine dioxide, hypochlorite and chlorite are commonly applied as bleaching agents and disinfectants. Highly oxidized inorganic forms of chlorine, such as perchlorate and chlorate, are also produced industrially. Whereas chlorate is used in the paper industry and as herbicide, perchlorate is a constituent of explosives and rocket fuels (Urbansky, 2002).

While the development and application of chlorine compounds has promoted agriculture, health care and industries, they have been main causes of environmental contamination and harm. The majority of persistent organic pollutants with severe impact on human, animal and environmental health listed by the international community at the Stockholm Convention on Persistent Organic Pollutants are chlorine pesticides (Hagen and Walls, 2005). However, the inglorious fame of such persistent and toxic man-made chlorine compounds in the environment has caused an underestimation of the widespread occurrence and diversity of natural chlorine compounds for a long time. In fact, organic and inorganic chlorine compounds may have been present on Earth long before life appeared (Gribble, 1998; Dasgupta et al., 2005; Plummer et al., 2006). In 1975, organochlorines were first recognized as part of the natural ecosystem, as opposed to their previously presumed anthropogenic origin (Lovelock, 1975). Since then, a large number of organochlorines produced through geogenic processes such as volcanic activity and forest fires, and biogenic sources, were reported (Gribble, 2010). Biogenic sources include volatile haloalkane formation by algae, fungi, bacteria, plants and termites (Moore, 2003), and biosynthesis of halogenated secondary metabolites by bacteria, fungi, plants and animals, especially in the marine environment (van Pée and Patallo, 2006; Gribble, 2010; Blunt et al., 2013). These natural sources of organohalogens greatly outnumber currently used synthetic halogenated compounds in diversity and in some cases in volume (Moore, 2003; Reineke et al., 2011). Over 5000 different natural organohalogens have now been identified, and about 200 additional compounds are discovered every year (Gribble, 2004, 2010). The formation of organochlorines was proposed to provide the producing microorganisms and/or their host organism a competitive advantage over predators or competitors (Engel et al., 2002).

The long-standing presence of chlorine compounds on Earth has driven the evolution of biological mechanisms that gain energy from the metabolism of organic and inorganic chlorine compounds. As such, chlorine compounds can be used as electron donors or terminal electron acceptors for microbial growth (Häggblom, 1992; Coates et al., 1999b; Hug et al., 2013). This feature has been applied for (bio)remediation studies of aquatic and terrestrial environments contaminated with chlorine compounds (Major et al., 2002; Lendvay et al., 2003; Stroo and Ward, 2008; Hermon et al., 2018).

**FIGURE 1:**
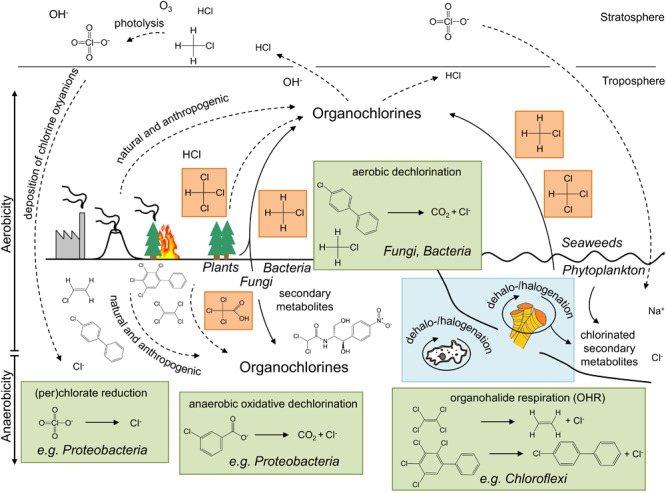
Selected chlorine compounds, their origin and (bio)transformation – a schematic overview of the global chlorine cycle. Representative microbial dechlorination processes and involved compounds are shown in green boxes. The most abundant biologically produced chlorine compounds are indicated in orange, whereas examples for local “microcycles” are highlighted in blue. Groups of (micro)organisms associated with a particular metabolism are indicated in italics. Solid and dotted lines illustrate biological and chemical processes, respectively.

The widespread occurrence of chlorine compounds in marine and terrestrial environments and various trophic food chains, as well as biotic and abiotic reactions converting chloride and chlorine compounds, suggest a substantial biogeochemical cycling of these compounds (Figure 1). While specific geochemical and biological aspects of the chlorine cycle are covered in excellent reviews (Neilson, 1990; Chaudhry and Chapalamadugu, 1991; Hardman, 1991; Häggblom, 1992; Bhatt et al., 2007), here we provide a comprehensive overview of the main microbial sources and sinks of chlorine compounds. We highlight the latest findings on microbial metabolism, and reflect on their co-occurrences and potential connections of catabolic and anabolic transformations of natural and synthetic chlorine compounds in nature. Finally, we discuss the knowledge gaps and future directions for approving or rejecting metabolic links between chlorinating and dechlorinating microbes.

## Microbial Chlorination

The vast majority of accessible chlorine on Earth is present in an inorganic form, such as NaCl. However, especially in the troposphere and stratosphere, chloromethane and to a lesser extent chloroform and other volatile chloroalkanes are major contributors to the atmospheric chlorine budget (Graedel and Keene, 1996). These volatile organochlorines enter the atmosphere mainly by emissions from oceans, peat bogs and forest soils (Gribble, 2004; Leri and Myneni, 2010). Natural organochlorines of higher complexity are generally found at trace concentrations in aquatic and terrestrial environments where they emerge as products of secondary metabolic pathways. Although natural organochlorines may be small in volume, they have much more structural variety than man-made organohalogens (Gribble, 2010). Most chlorine secondary metabolites have a chlorine atom (or multiple chlorine atoms) bound to phenol or pyrrole ring structures, but there are also many examples of chlorination at saturated ring structures in terpenoids and sterols. In addition, secondary metabolites are often decorated with chlorine at the terminal position of their saturated and unsaturated aliphatic side chains (Faulkner, 2000; Gribble, 2004; van Pée and Patallo, 2006). Chlorine secondary metabolites are of particular interest for pharmaceutical applications, as biological activity of secondary metabolites is often significantly altered if halogen atoms are introduced (Fenical and Jensen, 2006; van Pée and Patallo, 2006; Rastogi and Sinha, 2009; Gribble, 2015).

### Chlorinating Enzymes

The first halogenase discovered in the 1960s was a heme chloroperoxidase from the fungus *Caldariomyces fumago* (Hager et al., 1966). Subsequently, also vanadium-containing chloroperoxidases (Vilter, 1984) and cofactor-free chloroperoxidases (perhydrolases) (van Pée et al., 2000) were identified that are not phylogenetically related to heme chloroperoxidases (Xu and Wang, 2016). Chloroperoxidases rely on the presence of hydrogen peroxide to catalyze unspecific electrophilic halogenation reactions yielding organochlorines, such as mixtures of mono-, di- and trichloroacetic acid (Hoekstra, 2003) and polychlorinated haloalkanes such as chloroform (Hoekstra et al., 1998). It has been proposed that haloperoxidase biochemistry first evolved in cyanobacteria in response to the formation of hydrogen peroxide after the emergence of photosynthesis. Bromoperoxidases protect the organisms against reactive oxygen species (Ohsawa et al., 2001; Bernroitner et al., 2009), but it is not clear whether chloroperoxidases have a similar function.

**FIGURE 2:**
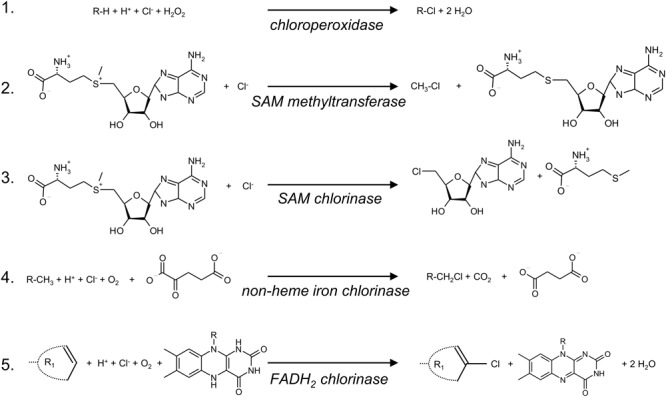
Schematic overview of enzymatic chlorination reactions. Reaction 1 is an electrophilic chlorination reaction of an alkyl group (R) catalyzed by heme- or vanadium-containing chloroperoxidases. Reactions 2 and 3 are nucleophilic chlorination reactions catalyzed by SAM-dependent methyl transferases that lead to the formation of mono substituted alkanes (2) or to the formation of 5′-chloro deoxyadenosine (3), which is the precursor of salinosporamide A. Reaction 4 is a radical chlorination at unactivated carbon centers and is catalyzed by an α-ketoglutarate-dependent non-heme iron chlorinase. Reaction 5 mainly leads to chlorination of tryptophan, phenol or pyrrole ring structures catalyzed by FADH_2_-dependent chlorinases. R1 represents a phenol, pyrrole or tryptophan moiety.

Since the initial discovery of chlorinating enzymes, biological chlorination has moved far beyond chloroperoxidases. Enzymes using *S*-adenosyl-L-methionine (SAM) as co-substrate are involved in halogenation via two different mechanisms (Figure 2). First, SAM-dependent methyl transferases, although strictly no chlorinases, facilitate the transfer of the methyl group from SAM through a nucleophilic attack of a chloride, leading to formation of chloromethane, the most abundant naturally produced organochlorine on Earth (Wuosmaa and Hager, 1990) (Figure 1). Second, a small family of SAM-dependent halogenases has been discovered in which the halide is bound in a hydrophobic pocket of the enzyme and displaces the L-methionine group from SAM producing halogenated adenosines as the first step in the biosynthesis of halogenated molecules (Eustáquio et al., 2008; Deng et al., 2014) (Figure 2). The SAM-dependent chlorinase from the marine actinobacterium *Salinispora tropica* is responsible for the chlorination reaction in the biosynthesis of the secondary metabolite salinosporamide A (NPI-0052) (Beer and Moore, 2007), which is in clinical development for treatment of myelomas (Fenical et al., 2009). The small family of SAM-dependent chlorinases is part of the DUF62 family, which contains over 100 proteins found in a broad range of bacteria and archaea (Deng and O’Hagan, 2008; Eustáquio et al., 2008). However, most of these proteins are SAM hydroxide adenosyltransferases to which the SAM-dependent halogenases may be evolutionarily closely linked (Deng et al., 2009; Xu and Wang, 2016).

The chlorination reaction in the synthesis of other secondary metabolites, such as jamaicamide A, which contains a vinyl chloride group and acts as a potent sodium channel blocker (Edwards et al., 2004), and barbamide, a chlorine compound with antifouling activity (Chang et al., 2002), is catalyzed by non-heme iron chlorinases (Vaillancourt et al., 2006; Van Pée, 2012). These α-ketoglutarate-dependent non-heme iron halogenases catalyze highly specific radical halogenations of unactivated carbon centers in amino acids that are linked to peptidyl carrier proteins (Figure 2). Recently, a new family of non-heme iron halogenases was discovered that do not require substrate coupled to a carrier protein but can chlorinate freestanding small molecules instead. Such an enzyme is responsible for the chlorination in the biosynthesis of welwitindolinones (Hillwig and Liu, 2014).

Flavin-dependent halogenases (FADH_2_-dependent halogenases) mediate the majority of the chlorination reactions and production of halogenated secondary metabolites. FADH_2_-dependent halogenases are primarily involved in the electrophilic halogenation of tryptophan, phenol and pyrrole moieties of secondary metabolites (van Pée and Patallo, 2006) (Figure 2). However, there are also a few examples of FADH_2_-dependent halogenases involved in the chlorination of aliphatic structures, for instance in the biosynthesis of chloramphenicol (Piraee et al., 2004). Chloramphenicol was isolated from the actinomycete *Streptomyces venezuelae*, and was the first chlorine secondary metabolite used as an antibiotic in clinical practice (Ehrlich et al., 1947). However, its use has declined due to bacterial resistance and toxic side effects (Balbi, 2004). Vancomycin is a currently used chlorine antibiotic (Levine, 2006), and the chlorination step in the biosynthetic pathway is mediated by an FADH_2_-dependent halogenase (van Pée, 2001).

### Chlorinating Organisms

**FIGURE 3:**
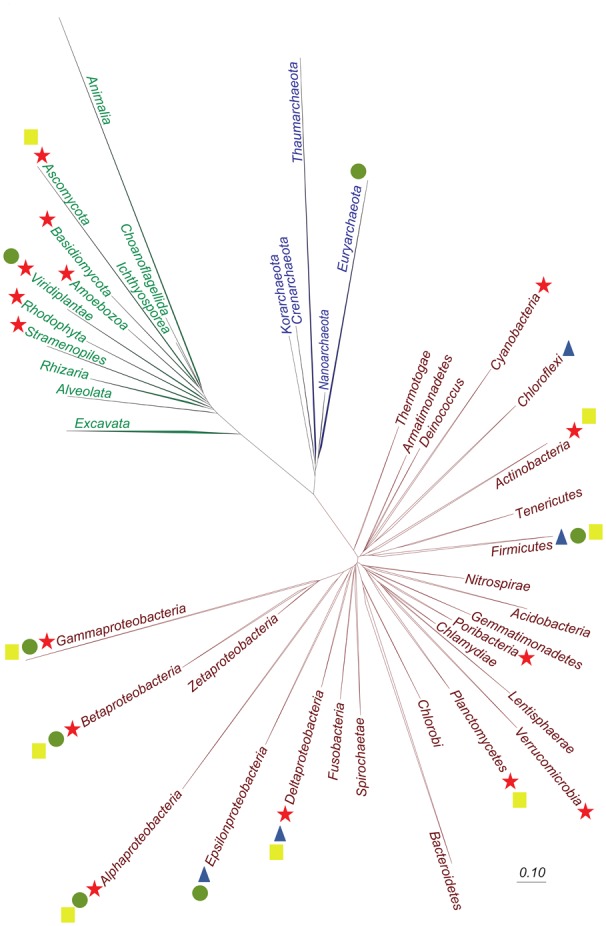
Tree of life showing the phyla or classes for which key enzymes of microbial (de)chlorination processes have been detected. The tree of life was prepared from the Silva SSU Ref NR 115 database. The bar illustrates a distance of 0.1 substitutions per site. Organisms that carry aerobic haloalkane dehalogenases are indicated by a yellow square. Reductive dehalogenase-baring organisms are shown by the blue triangle, organisms with chlorate- and perchlorate-reducing enzymes by the green circle and halogenase-producing organisms by the red star.

Biological chlorination or the presence of putative halogenase-encoding genes has been observed in a broad phylogenetic range of organisms (Figure 3). In the next section these organisms are reviewed.

#### Bacteria

In 1960, eight bacterial chlorine compounds were identified of which seven were of actinobacterial origin (Petty, 1961). Later, many more organochlorines were discovered in this class of organisms. For example, vancomycin and salinosporamide A are produced by the actinobacteria *Amycolatopsis orientalis* and *Salinispora tropica*, respectively (Levine, 2006; Fenical et al., 2009). Although members of the phylum Actinobacteria have remained the center of attention, a much broader phylogenetic spectrum of bacteria harbors putative FADH_2_-dependent halogenase-encoding genes including Alpha-, Beta-, Gamma- and Deltaproteobacteria, Cyanobacteria, Planctomycetes, Verrucomicrobia, and Poribacteria (Zehner et al., 2005; Wagner and König, 2012; Bayer et al., 2013; Öztürk et al., 2013) (Figure 3). Non-heme iron chlorinases were mostly detected in cyanobacteria (*Lyngbya majuscula* and *Oscillatoria spongeliae*) and are involved in the formation of e.g., barbamide and jamaicamide (Chang et al., 2002; Edwards et al., 2004). Furthermore, these enzymes occur in Betaproteobacteria (*Burkholderia* spp.), Gammaproteobacteria (*Pseudomonas syringae*) and Actinobacteria (*Streptomyces* spp., *Kutzneria* sp.) (Guenzi et al., 1998; Holden et al., 2004; Galonić Fujimori et al., 2007).

For a long time bacteria were not known for their capacity to also produce short-chain chloroalkanes, but at the end of the 20th century, common marine cyanobacteria such as members of the genera *Synechococcus* and *Chlorococcus* were identified as producers of chloromethane and a number of other single or polyhalogenated organohalogens (Scarratt and Moore, 1998; Brownell et al., 2010). Monosubstituted haloalkanes such as chloromethane appear to be by-products or ‘accidents’ of normal metabolism or may play a role as methylating agents in cell metabolism (Harper, 2000). More recently, also non-phototrophic marine bacteria, such as *Erythrobacter* and *Pseudomonas* strains, were found to be producers of chloromethane (Fujimori et al., 2012). Despite the release of chloromethane by common marine bacteria, the formation rates are low, and therefore marine bacteria are likely to contribute only a small fraction of the ocean-to-atmosphere flux of chloromethane (Brownell et al., 2010; Fujimori et al., 2012). Forest soils are known as a major source of volatile organochlorine compounds including chloromethane, chloroform and other alkylchlorides (Keppler et al., 2000). Volatile organochlorines in forest soils are formed during the decomposition of humic and fulvic acids via chlorine intermediates (Keppler et al., 2000; Laturnus et al., 2005). The relative contributions of abiotic and biotic processes to the formation of volatile organochlorines in forest soils remained unclear for long, but biological formation seems to be the predominant process (Bastviken et al., 2009). While the paradigm has been that biotic formation of volatile organochlorines in soil is mainly due to fungi, a recent study showed that the common and widespread soil bacterium *Sinorhizobium meliloti* may also be a significant producer of chloroform and tetrachloroethene (PCE) (Weigold et al., 2015). Moreover, metagenomic analyses revealed that genes encoding bacterial halogenases greatly outnumber genes encoding fungal halogenases in forest soil (Weigold et al., 2016).

#### Archaea

*Archaea* are not (yet) known for producing chlorine compounds. Only recently the first putative FADH_2_-dependent halogenase encoding gene was reported in the genome of the euryarchaeon *Methanotorris igneus* (Lucas et al., 2011) that was isolated from a shallow submarine hydrothermal vent (Burggraf et al., 1990). However, halogenating activity has yet to be confirmed.

#### Fungi

After the discovery of the first chlorinating enzyme in the fungus *Caldariomyces fumago*, fungi have remained a prolific biological source for the discovery of chlorinases and chlorine compounds. In fact, heme- and vanadium-dependent haloperoxidases with proven activity seem to be restricted to a specific group of plant-associated fungi and pseudofungi including the oomycete *Phytophthora infestans* (Wever and Hemrika, 2001; Bengtson et al., 2013). Chloroperoxidase-mediated chlorination of humic acids and lignin by fungi improves the bioavailability of these recalcitrant organic compounds and leads to the formation of halogenated aromatic compounds, such as 5-chlorovanillin and 2-chlorosyringaldehyde (Laturnus et al., 2005; Ortiz-Bermúdez et al., 2007). Similarly, wood-rotting fungi produce vast quantities of chloromethane through the activity of SAM-dependent methyl transferases (Spinnler et al., 1994; Watling and Harper, 1998; Gribble, 2004). In particular the genus *Phellinus* was held responsible for more than 80% of the chloromethane emission from tropical and subtropical forests (Watling and Harper, 1998). It remains to be seen if these high chloromethane emission volumes previously ascribed to fungi are partly due to chloromethane formation by bacteria sharing the same habitat.

Besides short-chain chlorine molecules, a number of chlorine secondary metabolites are produced by fungi, most notably caldariomycin, which contains a 1,1-dichlorocyclopentyl group from *Caldaromyces fumago* (Clutterbuck et al., 1940). Other examples include acrodontiolamide from *Acrodontium salmoneum* (de Gusmáo et al., 1993), which contains an unusual dichloromethyl ether moiety, and the tricyclic antifungal agent griseofulvin that was isolated from several *Penicillium* spp. (van Pée, 1996).

#### Unicellular Eukaryotes

Although white rot fungi are the best characterized source of the release of chloromethane, it is known for a long time that substantial quantities of chloromethane are released from the oceans. Seaweeds and salt marsh plants (not reviewed in this paper) are the best known marine sources for the release of chloromethane and other halomethanes (Harper, 2000), but phototrophic unicellular eukaryotes, such as *Phaeodactylum tricornutum, Emiliana huxleyi, Prorocentrum* sp., *Tetraselmis* sp. and *Phaeocystis* sp. also contribute to oceanic emissions. Their cumulative contribution has previously been estimated to represent less than 1% of the total oceanic chloromethane emission (Scarratt and Moore, 1998), however, the total chloromethane flux from the oceans is still under debate (Keppler et al., 2005).

For another unicellular eukaryote, the social amoeba *Dictyostelium discoideum*, chlorination is involved in the transformation of the single cell stage to forming a multicellular stage (Kay, 1992). An FADH_2_-dependent halogenase catalyzes the chlorination of the core polyketide in the biosynthesis of differentiation-inducing factor 1, which is an important regulator in stalk cell formation of *D. discoideum* (Neumann et al., 2010).

## Aerobic Metabolism of Organochlorine Compounds

### Degradation

Aerobic biodegradation studies on organochlorines have focused on synthetic compounds, and only a few studies have addressed the degradation of complex natural organochlorines. Aerobic degradation proceeds either via catabolic pathways that support microbial growth (Copley, 1998; Fetzner, 1998) or through cometabolism, i.e., fortuitous transformation by enzymes that serve other functions in the cell (Mattes et al., 2010). In general, the susceptibility of organochlorine compounds to aerobic degradation decreases if the number of chlorine atoms increases. Nevertheless, even several highly chlorinated synthetic chemicals such as trichloroacetate, pentachlorophenol and γ-hexachlorocyclohexane can serve as sole carbon and energy source for specific microorganisms, mostly bacteria (Fetzner, 1998; Harper, 2000; van Pée and Unversucht, 2003; Reineke et al., 2011). Naturally produced organochlorine compounds reported to be growth substrates for aerobic bacteria include chloromethane, chloroacetate, chlorobenzoates, and chlorophenols (Reineke et al., 2011). In turn, polychlorinated organic compounds such as PCE, hexachlorobenzene and polychlorinated dioxins and biphenyls are persistent in oxic environments, and stepwise anaerobic dechlorination processes discussed below are much more important for the metabolism of these compounds (Smidt and De Vos, 2004).

Numerous bacterial cultures utilizing chlorine compounds as carbon source or electron donor under aerobic conditions were isolated in the 1980s and 1990s, and catabolic pathways are summarized in several excellent reviews (Copley, 1998; Fetzner, 1998; Reineke et al., 2011). In most cases, aerobic organisms produce dedicated dehalogenases that selectively act on carbon-chlorine (or carbon-halogen) bonds (Kurihara and Esaki, 2008). Detailed biochemical analysis of catabolic pathways and improvement of key enzymes by protein engineering occasionally made it possible to engineer organisms for degradation of recalcitrant organochlorine compounds, as exemplified with recombinant *Pseudomonas* strains degrading the toxic compound 1,2,3-trichloropropane in a continuous flow bioreactor (Samin et al., 2014; Gong et al., 2017).

**FIGURE 4:**
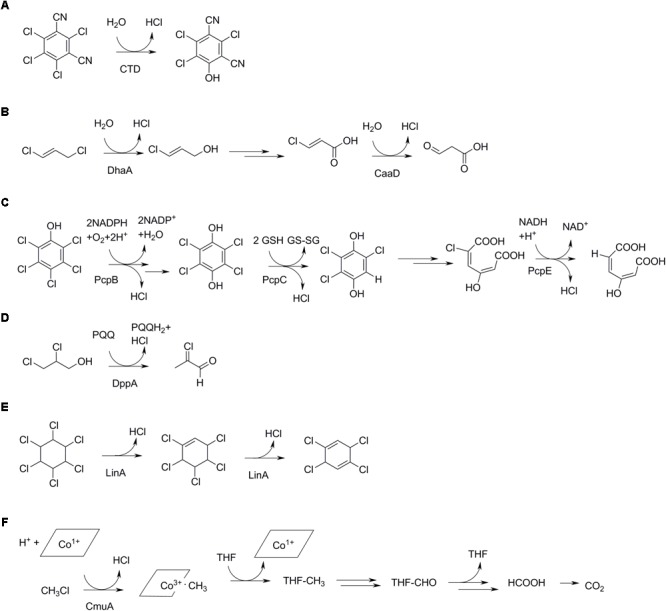
Productive routes for utilization of selected organochlorine compounds in bacteria. **(A)** Initial step in chlorothalonil degradation, catalyzed by a dehalogenase (CTD) (Liang et al., 2011). **(B)** 1,3-dichloropropene degradation by *Pseudomonas pavonaceae* (Poelarends et al., 1998). The initial dehalogenase (DhaA) is found in haloalkane-degrading bacteria from different geographic regions, whereas the second dehalogenase (CaaD) is unrelated and rare (Poelarends et al., 2000). **(C)** Pentachlorophenol metabolism by an initial monooxygenase (PcpB) reaction and two reductive reactions, the first one (PcpC) involving a glutathione transferase, and the second one (PcpE) involving an NADH-dependent reductase (Kiefer and Copley, 2002; Hlouchova et al., 2012). **(D)** Degradation of 2,3-dichloropropanol via the highly reactive intermediate 2-chloroacrolein (Arif et al., 2012). **(E)** The insecticide hexachlorohexane undergoes two initial dechlorinations by elimination by a dehalogenase (LinA). **(F)** Aerobic methylchloride metabolism involving a corrinoid cofactor of the methyltransferase (CmuA) and tetrahydrofolate (THF) as methyl acceptor (Stourman et al., 2003).

Most of these studies on microbial dechlorination were inspired by environmental contamination with pesticides, chlorinated solvents, industrial chemicals like PCBs and waste compounds such as chlorinated dioxins. A recent example is a study on the growth-supporting biodegradation of the fungicide chlorothalonil (Liang et al., 2010) (Figure 4A), which undergoes initial hydrolytic dechlorination without prior activation of the aromatic ring. Current work often explores the fate of emerging contaminants related to compounds that are in everyday use, like chlorinated household products and pharmaceuticals. Here, a recent example is the disinfectant triclosan, which can be degraded by a strain of *Sphingomonas* that first oxygenates the monochlorinated ring, then cleaves the ring and produces 2,4-dichlorophenol and probably a dihydroxylated chloromuconic acid (Mulla et al., 2016).

Both in case of growth-supporting routes and cometabolic dechlorination, the actual chloride-release step is often preceded by other reactions, especially oxygenation and dehydrogenation. For example, oxidative degradation of the herbicide 2,4-D in bacteria is initiated by a α-ketoglutarate-dependent dioxygenase that cleaves the ether bond and a dioxygenase reaction that splits the aromatic ring forming a dichloromuconic acid. Dechlorination occurs after that by intramolecular substitution leading to lactone formation and by reductive dechlorination (Kumar et al., 2016). Whereas biochemical studies on isolated cultures could give a biased or incomplete view of processes occurring *in situ*, the environmental relevance of such oxygenation pathways is supported by proteome analysis, which identified key oxygenases in 2,4-D-spiked microcosms and groundwater samples contaminated with chlorobenzene (Benndorf et al., 2007).

### Dehalogenase Mechanisms

The widespread release of chlorine pesticides and solvents in the environment has triggered evolution and/or proliferation of genes encoding enzymes specifically breaking carbon-chlorine bonds. Dozens of such dehalogenases have been identified, and crystal structures are available of a range of mechanistic classes. Many dechlorinating enzymes possess a distinct halogen/halide binding site, which contributes to catalysis by stabilizing the negative charge that develops in the transition state leading to carbon-chlorine bond cleavage. Predominant reactions catalyzed by aerobic dehalogenases are substitution (Figures 4A,B), oxidation (Figure 4C), dehydrogenation (Figures 4C,D), and elimination (Figure 4E) (Copley, 1998; Fetzner, 1998; van Pée and Unversucht, 2003). Oxidation and reduction may involve enzymes that mechanistically do not cleave the carbon-chlorine bond but render them labile, like in cometabolic transformations (see below).

Substitution with water by a hydrolytic dehalogenase often follows a two-step mechanism with initial displacement of the chlorine through nucleophilic attack by an active site aspartate, followed by cleavage of the ester intermediate by water. This mechanism occurs in hydrolytic dehalogenases acting on chloroalkanes, which belong to the α/β-hydrolase fold enzymes, and in haloacetate dehalogenases which are related to phosphatases and were classified as members of the HAD (haloacid dehalogenase) superfamily (Janssen, 2004; Kurihara and Esaki, 2008). The haloalkane dehalogenases are likely involved in hydrolysis of natural 1-chloro-*n*-alkanes but certainly also in the metabolism of synthetic organochlorines such as 1,2-dichloroethane, chlorinated ethers, and 1,3-dichloropropene. The latter compound has been extensively used in agriculture to combat potato nematodes, resulting in the direct large-scale field application of a chlorinated hydrocarbon. In a *Pseudomonas* strain obtained from a field showing accelerated degradation of 1,3-dichloropropene, the 1-chloro-3-propenol that is formed by the initial hydrolysis reaction is converted to 3-chloroacrylic acid (Figure 4B). This intermediate undergoes further dechlorination by a different specific dehalogenase that belongs to the 4-OT superfamily of proteins, which typically catalyze hydration and isomerization reactions on double bonds in carboxylic acids (Poelarends et al., 2001).

Other substitutions involve the sulfhydryl of glutathione or an enzyme cysteine residue. The former characterizes the dehalogenases involved in dichloromethane metabolism in *Methylobacterium* and *Hyphomicrobium* (Stourman et al., 2003), whereas a glutathione and an enzyme sufhydryl are employed by a glutathione transferase catalyzing a step in pentachlorophenol degradation by *Sphingobium chlorophenolicum* (Warner et al., 2005). In the latter case, dechlorination starts with a monooxygenase reaction (Hlouchova et al., 2012) after which the product tetrachlorohydroquinone is further dechlorinated by a glutathione transferase (Kiefer and Copley, 2002) (Figure 4C). The enzyme catalyzes a remarkable net reduction of the substrate in this aerobic degradation pathway by converting tetrachlorohydroquinone to trichlorohydroquinone with formation of oxidized glutathione (disulfide). Whereas nucleophilic substitutions on aromatic rings are scarce, in this case the aromatic ring is relatively susceptible due to the presence of electron-withdrawing hydroxyl and chlorine groups.

Methyl chloride, produced naturally as described above, can be degraded aerobically by various methylotrophic soil organisms of the genera *Hyphomicrobium* and *Methylobacterium*. Metabolism starts with transfer of the methyl group to the corrinoid cofactor of the methyltransferase by an enzyme designated CmuA (chloromethane utilization) (Figure 4F). Subsequent methyl transfer from the corrinoid protein to tetrahydrofolate (CmuB) and oxidation steps yield CO_2_ (Vannelli et al., 1998; Borodina et al., 2005) (Figure 4F).

### Cometabolic Dechlorination

In contrast to growth-supporting metabolism, cometabolism is usually caused by a broad range of enzyme specificities. Enzymes that do not specifically cleave carbon-halogen bonds may catalyze or facilitate dechlorination by generating labile structures that undergo spontaneous (chemical) decomposition in aqueous medium (Suttinun et al., 2012; Nzila, 2013). Examples of labile compounds generated through unspecific enzymes are gem-chlorohydrins or chlorinated epoxides formed by bacterial monooxygenases (van Hylckama et al., 1996; Mattes et al., 2010; Cappelletti et al., 2012). Aerobic transformation by broad-specificity monooxygenases is well documented for various organisms including methanotrophs (methane monooxygenase), nitrifiers (ammonia monooxygenase), butane-oxidizing bacteria and toluene degraders (Hamamura et al., 1997; Mattes et al., 2010). Cometabolic transformation activities can be employed for *in situ* bioremediation of polluted sites by stimulating the growth and activity of oxidizing bacteria through injection of oxygen and ethene or methane; the latter may also originate from natural sources (Coleman et al., 2002). Indeed, high numbers of ethene- and methane-oxidizing organisms occur in biostimulated vinyl chloride-contaminated groundwater plumes (Mattes et al., 2015; Findlay et al., 2016; Atashgahi et al., 2017b). Predominant bacteria include members of *Mycobacterium, Nocardioides* and *Pseudomonas*. Cometabolism may also involve fungal enzymes. For example, white-rot basidiomycetes produce extracellular lignolytic enzymes capable of oxidizing organochlorines. Transformations that were studied include dechlorination of PCBs by laccase produced by *Pycnoporus cinnabarinus* (Schultz et al., 2001), and the same organism can transform triclosan (Hundt et al., 2000).

### Origin and Distribution of Dechlorinating Enzymes

Most dehalogenases acting on carbon-halogen bonds possess a specific halide-binding site which can be identified by structural studies, showing that they are not enzymes evolved for other reactions that fortuitously also catalyze dehalogenation. In case of atrazine- and 1,2-dichloroethane-degrading bacteria there is clear evidence that the initial dehalogenases have recently evolved from a closely related ancestor to obtain activity with synthetic compounds (Seffernick and Wackett, 2001; Janssen et al., 2005; Scott et al., 2009). An atrazine-degrading *Pseudomonas* strain produces a metal-containing aromatic dehalogenase that likely has evolved by a few mutations from a deaminating enzyme. Similarly, the predecessor of the 1,2-dichloroethane degrading dehalogenase in *Xanthobacter autotrophicus* likely underwent just a few cap domain mutations, including an insertion which appears as a short repeat in the gene, to evolve from a debrominating into a dechlorinating enzyme (Janssen et al., 2005). On the other hand, haloalkane dehalogenases are also widely present in organisms and ecosystems with no track record of degrading synthetic organohalogens (Hesseler et al., 2011; Chaloupkova et al., 2014; Guan et al., 2014; Carlucci et al., 2016; Weigold et al., 2016), suggesting a role in the metabolism of natural halogenated compounds. Another intriguing observation is that identical dehalogenase genes are repeatedly obtained by classical enrichment using synthetic compounds whereas these genes do not appear in general genomic databases (Poelarends et al., 1998; Poelarends et al., 2000; Govender and Pillay, 2011; Munro et al., 2016), indicating that enrichment identifies a widely distributed subpopulation of dehalogenase-producing organisms that does not represent the majority of the dehalogenase activity present in natural environments. These specific dehalogenase genes may be widespread: identical 1,2-dichloroethane dehalogenase (DhlA) genes were discovered in the Netherlands, South Africa, Korea, and Australia. However, they are located on different plasmids and associated with different insertion sequences, illustrating the high mobility of the genes (Song et al., 2004; Munro et al., 2016). The mechanism of distribution and the ancestry of 1,2-dichloroethane isolates remain unknown. However, the detection in independent microbial isolates of identical haloalkane dehalogenase genes which have no close homologs in (meta) genome sequences from organisms or samples with no history of 1,2-dichloroethane metabolism shows that classical selective enrichment accesses a part of natural biodiversity (and dehalogenation potential) that remains undisclosed when environmental samples are explored by genomic or proteomics studies only. Nevertheless, the use of enrichment cultures may cause a biased view on the actual diversity of bacteria degrading organochlorine compounds, as was shown for chloromethane-degrading bacteria, where the diversity discovered by genetic methods by far exceeded the diversity found by enrichment cultivation (Borodina et al., 2005; Cox et al., 2012).

The global distribution of the dechlorinating enzymes suggests a role in the degradation of natural compounds. The identity of the original substrates remains obscure, but the number of possibilities is enormous in view of the large diversity of organochlorines discussed above. Whereas evidence for recent evolution of catabolic activities at the enzyme level remains scarce, there is ample evidence for distribution of catabolic activities among organisms exposed to such compounds (Munro et al., 2016). Indeed, the observed residence of catabolic genes on transmissible plasmids, their association with transposons, and the localization of gene clusters on genomic islands are remarkable signs of distribution via horizontal gene transfer and assembly by natural genome shuffling (Van der Meer et al., 1992; Poelarends et al., 2000; Liang et al., 2011).

### Recalcitrance

Many synthetic chlorine chemicals are poorly degraded under aerobic conditions which is attributed in the first place to a lack of efficient catabolic pathways rather than to thermodynamic causes. Thus, free energy differences for aerobic mineralization of highly chlorinated compounds (chloroform, PCE) are negative indicating thermodynamic feasibility, yet no organisms are known for their aerobic mineralization (Dolfing and Janssen, 1994). Thus, biochemical causes prevent degradation and gain of energy. The foremost important factor is the lack of enzymes that can cleave the carbon-chlorine bonds in the original substrate or in key intermediates. Examples are highly chlorinated ethanes and propanes, including 1,1,1-trichloroethane and 1,2,3-dichloropropane. The absence of complete productive metabolic routes prevents proliferation by selective growth.

Substrate toxicity and formation of toxic intermediates can also prevent growth or rapid metabolism and, in case of toxic products, select for organisms that lack the capacity to transform chlorine compounds (Nikel et al., 2013). Acylchlorides, chlorinated aldehydes, and chloroepoxides are strong alkylating agents that may inactivate different biomolecules including enzymes and DNA. Enzyme inactivation may result in accumulation of reactive oxygen species, causing further damage to the cell, as was observed in 1,3-dichloropropene degrading *Pseudomonas* (Nikel et al., 2013). Although the reactions have been scarcely studied, it is interesting that catabolic gene clusters for xenobiotic compounds often include genes encoding glutathione transferases, enzymes which are involved in detoxification (Fortin et al., 2006). Indeed, expression of glutathione transferase was described to increase the cometabolic transformation of *cis-*dichloroethene epoxide (Rui et al., 2004).

## Anaerobic Metabolism of Organochlorine Compounds

### Anaerobic Dechlorination

Most subsurface environments such as aquifers, deeper soil layers and sediments are oxygen-depleted and conducive to anaerobic respiration where the oxidation of organic matter is coupled to reduction of a wide array of inorganic and organic electron acceptors including organochlorines (Leys et al., 2013). Under anoxic conditions, organochlorines with few chlorine substituents can be used as carbon or energy source by different bacteria such as denitrifying proteobacterial isolates growing on halobenzoates (Song et al., 2000), 2-chloroethanol (Dijk et al., 2003) or 1,2-dichloroethane (Dinglasan-Panlilio et al., 2006). Some homoacetogenic bacteria can utilize aliphatic chlorinated methanes such as chloromethane (*Acetobacterium dehalogenans*) (Traunecker et al., 1991) and dichloromethane *(Dehalobacterium formicoaceticum)* (Mägli et al., 1998) as carbon and energy source. Furthermore, fermentative growth on dichloromethane occurs in enrichment cultures containing *Dehalobacter* spp. (Lee et al., 2011; Justicia-Leon et al., 2012). These were the first reports showing fermentative growth by *Dehalobacter* spp., known to be restricted to organohalide respiration (OHR) as the sole metabolism. Phototrophic purple non-sulfur bacteria belonging to *Rhodospirillum* and *Rhodopseudomonas* spp. were reported to degraded halocarboxylic acids or 3-chlorobenzoate under anoxic conditions (Van der Woude et al., 1994; McGrath and Harfoot, 1997).

A new type of haloacids degradation under chlorate-reducing conditions was recently documented. *Pseudomonas chloritidismutans* AW1^T^ was shown to couple degradation of haloacids to chlorate reduction during which oxygen was produced by chlorite dismutation and further used (Peng et al., 2017). Although haloacid degradation was achieved by hydrolytic haloacid dehalogenases rather than using the produced oxygen, this was the first report showing concurrent degradation of an organic and inorganic chlorine compound by a single bacterium (Peng et al., 2017), and the first demonstration of an “intra-aerobic” pathway for organochlorine degradation. Such intra-aerobic microbes can derive oxygen from inorganic oxo-compounds such as nitrate or chlorate for oxygen-dependent degradation of hydrocarbons (Oosterkamp et al., 2013; Atashgahi et al., 2018b).

Some early reports also exist on complete mineralization of chlorine compounds under iron-reducing or methanogenic conditions (Bradley and Chapelle, 1996, 1999). However, no isolates have been obtained, and subsequent studies pointed toward aerobic degradation under hypoxic conditions (Bradley and Chapelle, 2011), possibly due to oxygen penetration during sampling of the microcosms. Overall, knowledge about the anaerobic degradation of organochlorines as the sole carbon and energy source remains limited. In contrast, extensive studies have been done focusing on dechlorination of organochlorines as electron acceptors. During respiratory reductive dechlorination, also termed OHR, the sequential removal of chlorine substituents is coupled to chemiosmotic energy conservation (Hug et al., 2013; Leys et al., 2013) (Figure 1). This process is mediated by organohalide-respiring bacteria (OHRB) that currently belong to the phyla Chloroflexi, Firmicutes, and Proteobacteria (Atashgahi et al., 2016) (Figure 3).

### Dehalogenase Mechanisms

Rather than through reductive dechlorination, anaerobic degradation of chloromethane occurs via a corrinoid-dependent methyl transfer system (Traunecker et al., 1991), and hence is similar to the mechanism employed by aerobic methylotrophic bacteria capable of assimilating chloromethane (Figure 4F). A similar methyl transferase system was employed by *D. formicoaceticum* converting dichloromethane to formate, acetate and chloride (Mägli et al., 1998). It is interesting that a methyl transfer system is not only used for dechlorination by aerobic and anaerobic bacteria, but also for halogenation reactions mediated by the SAM-dependent halogenases. However, different cofactors are applied, i.e., a corrinoid for dechlorination and SAM for halogenation.

**FIGURE 5:**
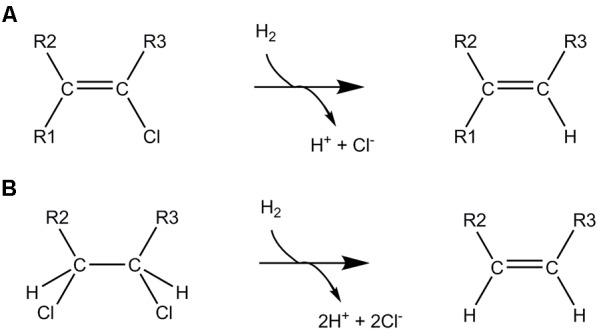
Anaerobic dehalogenation mechanisms. Reductive dechlorination by **(A)** hydrogenolysis, **(B)** dihaloelimination.

Reductive dechlorination is mediated by either hydrogenolysis (Figure 5A), where a chlorine substituent is replaced with a hydrogen atom, or dihaloelimination (Figure 5B), where removal of two chlorine substituents from adjacent carbon atoms is accompanied by the formation of an additional bond between the carbon atoms (Fincker and Spormann, 2017). As such, dihaloelimination is restricted to organochlorines with vicinal chlorinated carbon atoms. Bacteria belonging to the genus *Dehalogenimonas* (Chloroflexi) are particularly known for their dihaloelimination metabolism and energy conservation (Moe et al., 2016). The key enzymes in OHR are membrane-associated reductive dehalogenases encoded by *rdhAB* genes. The *rdhA* gene encodes the catabolic subunit and *rdhB* codes for a putative membrane anchor. The catabolic subunit generally contains two C-terminal iron-sulfur clusters, a corrinoid cofactor (usually cobalamin, B_12_, but also other variants were observed) and an N-terminal TAT (twin-arginine translocation) signal peptide (Fincker and Spormann, 2017; Schubert et al., 2018). The TAT signal is necessary for secretion of the mature reductive dehalogenase protein across the cell membrane where it is located at the exocytoplasmic face of the cytoplasmic membrane.

A new type of reductive dehalogenase was recently found in aerobic bacteria. It lacks the TAT translocation signal and was proposed to be located in the cytoplasm (Chen et al., 2013; Payne et al., 2015). Therefore, rather than a “respiratory” reductive dehalogenase used for energy conservation, this new group of enzymes was predicted to act as “catabolic” reductive dehalogenase. This catabolic function would be used to convert an organohalogen to non-halogenated carbon scaffold available for downstream heterotrophic degradation. Such reductive dehalogenase genes lacking the TAT signal were found in metagenomic analysis of marine sediments and may confer ecological advantage by facilitating further catabolism of the organic carbon resources, that are otherwise locked as organohalogens (Atashgahi et al., 2018a).

### Cometabolic Reductive Dechlorination

Cometabolic reductive dechlorination of organochlorines can be mediated by metal-containing porphyrins such as vitamin B_12_, cofactor F_430_ and hematin that contain a cobalt, nickel and iron atom, respectively, in their active center (Van Eekert et al., 1999). Such protein-bound tetrapyrrole cofactors are found in anaerobes like methanogens, acetogens, or sulfate-reducing bacteria, some of which are involved in cometabolic alkyl reductive dechlorination reactions (Holliger et al., 2004). Cometabolic reductive dechlorination is usually slow compared to OHR and does not often lead to complete dechlorination to non-dechlorinated products. Due to the fortuitous transformation of a compound by enzymes used by organisms for other beneficial purposes, the involvement of cometabolism in reductive dechlorination reactions is hard to discern. For example, mixed microbial communities dechlorinating organochlorines showed lower growth rates as compared to known OHRB, likely due to involvement of yet unknown OHRB or cometabolic dechlorination (Schneidewind et al., 2014; Lu et al., 2017).

### Origin and Distribution of Reductive Dehalogenases

The evolutionary origin of reductive dehalogenase genes is not known. Non-oxygenolytic reductive dechlorination processes have probably developed in the originally oxygen-free atmosphere on Earth. However, assuming that microbes harboring these enzymes feed on the natural organochlorines (Krzmarzick et al., 2012; Atashgahi et al., 2018a), and the fact that nearly all chlorinating enzymes use oxygen or peroxides as reactants (Field, 2016), the origin of the reductive dehalogenases should be after the “Great Oxidation Event” over two billion years ago (Zinder, 2016). As a common genomic trait, most *rdhAB* genes reside on mobile genetic elements, or are flanked by genes that suggest a mobile history (Futagami et al., 2006; McMurdie et al., 2009; Buttet et al., 2013), indicating horizontal transfer.

Using PCR-based methods, *rdhA* genes are commonly found in contaminated sites (Hug and Edwards, 2013; Atashgahi et al., 2017b), implying a cause-and-effect relationship between the occurrences of these genes and organochlorine contaminants. With the recent advances in metagenomic sequencing, environmental distribution of *rdhA* genes has been extended to environments not known to be contaminated with anthropogenic organohalogens such as a remote meromictic lake, Arctic tundra and forest soils, and marine sediments (Biderre-Petit et al., 2016; Weigold et al., 2016; Starnawski et al., 2017; Zlamal et al., 2017). It is likely that natural organohalogens accounting at these environments are substrates of the microbes harboring *rdhA* genes (Krzmarzick et al., 2012; Atashgahi et al., 2018a). In particular, *rdhA* genes (Kawai et al., 2014; Marshall et al., 2018) and their transcripts (Zinke et al., 2017) were found in deep marine sediments of up to hundred meters of depth, and spanning up to one million years of age since deposition. This shows active halogen cycling in deep marine sediments and further attests the ancient origin of *rdhA* genes. Interestingly, *rdhA* genes were also found in the genomes of novel archaeal groups proposed as the origin of eukaryotic cells (Zaremba-Niedzwiedzka et al., 2017). However, functionality of the enzymes encoded by these genes remains to be verified. More recent (meta)genomic mining even revealed the presence of *rdhA* genes in microbial isolates retrieved from the human gut and metagenomic datasets obtained from the (non-)human gut (Atashgahi et al., 2018d). Exposure to various halogenated compounds in food, water, pharmaceuticals and personal care products likely promoted acquisition of *rdhA* genes by the gut microbiota.

### Recalcitrance

OHRB are fastidious microbes that are dependent on other microbial community members for electron donors and organic cofactors. On the other hand, many subsurface environments are oligotrophic, and therefore the natural attenuation of organochlorine contaminants is often slow (Schneidewind et al., 2014). A straightforward and affordable approach for enhancing *in situ* reductive dechlorination is through the injection of organic carbon sources and/or bioaugmentation with enrichment cultures at contaminated sites. This usually leads to enhanced microbial activity and highly reducing environments that can in turn enhance reductive dechlorination by a suit of respiratory, cometabolic and abiotic reactions (He et al., 2015; Atashgahi et al., 2017b). The appearance of OHRB following biostimulation indicates their presence at the contaminated sites, owing to their resistance to the contaminant and/or their organohalide-respiratory metabolic potential (Atashgahi et al., 2018c). However, even after repeated rounds of biostimulation and bioaugmentation, complete dechlorination may not be achieved. A broad range of factors, including, e.g., presence of (non-)chlorinated co-contaminants, lack of key nutrients, competition with local microbial community members, and spatial and temporal subsurface heterogeneity, may restrict the activity of OHRB (Atashgahi et al., 2018c).

Far less information is available about the fate of organochlorine compounds from natural sources. However, the fact that these compounds can be found in higher food webs such as marine mammals (Teuten and Reddy, 2007) and ultimately humans (Wang et al., 2012) shows their recalcitrance, owing to their persistence and bioaccumulative properties. Similarly, chlorine compounds originating from pharmaceuticals and personal care products that usually occur at micropollutant concentrations (μg-ng range) are widely found in the environment, indicating their recalcitrance to microbial metabolism (Atashgahi et al., 2018d). It remains to be elucidated if such trace concentrations can induce and sustain any microbial catabolic activities in the environment.

## Microbial Metabolism of Chlorine Oxyanions

### Chlorine Oxyanions: Origin, Chemistry and Microbial Degradation

The chlorine oxyanions perchlorate (ClO_4_^-^) and chlorate (ClO_3_^-^) contain chlorine in an oxidized form (+VII and +V). Their salts are highly soluble in water, and the high redox potential (ClO_4_^-^/ClO_3_^-^ *E*_0_′ = +0.788 V; ClO_3_^-^/ClO_2_^-^ *E*_0_′ = +0.709 V; ClO_2_^-^/Cl^-^ *E*_0_′ = +1.199 V) makes them ideal electron acceptors for microorganisms. Besides anthropogenic sources, natural formation and deposition of chlorine oxyanions have contributed to their environmental occurrence (Parker, 2009; Catling et al., 2010; Rao et al., 2010). Of all chlorine oxyanions, perchlorate is chemically the most stable compound. However, naturally deposited perchlorate does not accumulate on Earth (except in hyperarid areas such as the Atacama desert) due to its microbial reduction on a global scale (Rikken et al., 1996). Complete reduction of chlorine oxyanions results in the formation of chloride (Cl^-^) (Figure 1) (Rikken et al., 1996). Contrarily to perchlorate and chlorate, more reduced chlorine oxyanions like chlorine dioxide (ClO_2_), chlorite (ClO_2_^-^) and hypochlorite (ClO^-^) are very reactive antimicrobials and thus induce prokaryotic defense mechanisms upon exposure. It was speculated that, due to the wide phylogenetic distribution of halo- (and more precisely chloro-) peroxidases, almost all microorganisms could be exposed to reactive chlorine species in their environment (Gray et al., 2013).

In the early 20^th^ century, microbial reduction of chlorine oxyanions was reported (Aslander, 1928), but the first axenic perchlorate-reducing bacterium was described 50 years later (Korenkov et al., 1976). The majority of currently known chlorate- and perchlorate-reducing bacteria are proteobacterial (predominantly betaproteobacterial) facultative anaerobes (Figure 3). Their physiology was comprehensively reviewed (Coates et al., 1999b; Coates and Achenbach, 2004; Bardiya and Bae, 2011; Nilsson et al., 2013; Youngblut et al., 2016b). More recently, members of the Gram-positive Firmicutes, the hyperthermophilic archaea *Archaeoglobus fulgidus* and *Aeropyrum pernix* (Liebensteiner et al., 2015) and some members of the archaeal *Halobacteriaceae* were also reported to couple the reduction of chlorine oxyanions to growth (Okeke et al., 2002; Balk et al., 2008; Balk et al., 2010; Liebensteiner et al., 2013; Oren et al., 2014) (Figure 3).

### Enzymes Reducing Chlorine Oxyanions

**FIGURE 6:**
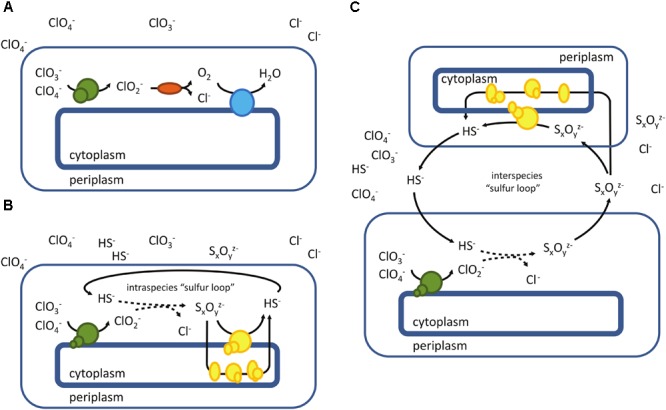
Microbial reduction of chlorine oxyanions – a schematic overview. **(A)** “classical” perchlorate reduction involving a functional chlorite dismutase (Cld, mainly found in mesophilic *Proteobacteria*), **(B)** reduction of chlorine oxyanions in the absence of Cld, employing an “intraspecies sulfur loop” (as found in *A. fulgidus*), **(C)** potential syntrophic reduction of chlorate and perchlorate, involving interspecies sulfur transfer. Perchlorate-reducing enzymes are depicted in green, chlorite dismutase in red and terminal oxidase in blue. Enzymes involved in the reduction of oxidized sulfur compounds (e.g., S^0^, S_2_O_3_^2-^, S_4_O_6_^2-^, etc.) are shown in yellow.

Biological perchlorate reduction commonly involves two enzymes, perchlorate reductase and chlorite dismutase (Cld) (Rikken et al., 1996). Perchlorate reductase catalyzes the first two reduction steps, from perchlorate to chlorate and from chlorate to chlorite. Chlorite is subsequently disproportioned by chlorite dismutase to chloride and molecular oxygen (Figure 6A). Some microorganisms cannot use perchlorate but still reduce chlorate to chlorite followed by chlorite disproportionation; chlorate reduction is mediated by chlorate reductases (Clr) that differ from perchlorate reductases (Pcr) in evolutionary, genetic and enzymatic aspects (Kengen et al., 1999; Danielsson Thorell et al., 2003; Bender et al., 2005; Melnyk et al., 2011; Clark et al., 2013). Horizontal gene transfer seems to have played an important role in dissemination of both chlorate and perchlorate reduction traits (Melnyk et al., 2011; Clark et al., 2013; Melnyk and Coates, 2015; Youngblut et al., 2016b). Pcr and Clr are periplasmic oxidoreductases that belong to the DMSO reductase family type II, and resemble nitrate reductases and other oxidoreductases containing molybdopterin as a cofactor (Nilsson et al., 2013). Several enzymes within the respective enzyme family have demonstrated reactivity toward more than one substrate. Nar-type nitrate- and selenate reductases are closely related to Pcr and Clr, and both enzymes can reduce chlorate in addition to nitrate or selenate (Martinez-Espinosa et al., 2007; Marangon et al., 2012). The structure of the active center of Pcr was elucidated and compared with the active center of Nar-type nitrate reductase. This indicated specific adaptations in the substrate access tunnel of Pcr toward oxyanions, and provided an explanation for the lower affinity of Nar-type nitrate reductase toward these substrates (Youngblut et al., 2016a). Other molybdenum-enzymes like DMSO reductase and trimethylamine N-oxide (TMAO) reductase have also been reported to reduce chlorate, whereas the periplasmic Nap-type nitrate reductase lacks this trait (Yamamoto et al., 1986; Weiner et al., 1988; Bell et al., 1990). Hence, there is a considerable range of molybdenum-enzymes that can reduce chlorate, albeit in most cases with lower catalytic efficiency. The activity of above mentioned enzymes toward perchlorate has been barely tested.

### Diversity of Chlorite Dismutase

Chlorite dismutase is a key enzyme for chlorate- and perchlorate-reducing microorganisms for disproportionation of the toxic intermediate chlorite and complete reduction of chlorine oxyanions coupled to growth. The immense diversity of Cld and Cld-like proteins and their presence throughout the tree of life have received particular attention over the last few years (Maixner et al., 2008; Mlynek et al., 2011). Recent reviews on functionally efficient Cld and Cld-like proteins discussed evolutionary, biochemical and biophysical aspects as well as their biotechnological potential (Hofbauer et al., 2014; DuBois and Ojha, 2015; Wang and Coates, 2017). The discovery of a Cld in the nitrite-oxidizing *Nitrospira defluvii* (Maixner et al., 2008) was the first example of a functional Cld in a bacterium incapable of chlorine oxyanion reduction. This Cld is similar to the group of Clds from known chlorate and perchlorate reducers (lineage I). The structurally different Cld of *Nitrobacter winogradskyi* (Mlynek et al., 2011), *Klebsiella pneumoniae* (Celis et al., 2015) and *Cyanothece* sp. (Schaffner et al., 2015), represent a novel lineage of Cld (lineage II) in microorganisms incapable of growth by chlorine oxyanion reduction. This Cld subtype may detoxify the intracellular chlorite formed by the reduction of environmental chlorate (and probably also perchlorate) through endogenous nitrate-reducing pathways or may play a yet unforeseen role in the chlorine cycle (Celis et al., 2015).

The distribution of *cld* and *cld*-like genes is also influenced by horizontal gene transfer (Maixner et al., 2008). Of more than 3,200 complete genomes available via the Integrated Microbial Genomes (IMG) database (Markowitz et al., 2012), we found that around 660 genomes contain genes encoding Cld family proteins (pfam06778); embracing a broad diversity of (micro)organisms. For instance, *Haloferax meditteranei* and other members of the Halobacteriaceae that are able to grow anaerobically with chlorate and perchlorate (Okeke et al., 2002; Oren et al., 2014) have genes encoding Cld-like proteins. Interestingly, mutation studies with bacteria that use chlorine oxyanions as terminal electron acceptors showed that the reduction and dismutation steps may be distributed over two microorganisms and not necessarily be combined in a single microorganism as generally thought (Clark et al., 2016). This is also supported by metagenomic analysis of enrichment cultures (Barnum et al., 2018). Even though the diversity of functional Cld goes beyond known chlorate- and perchlorate-reducing bacteria, not all *cld*-like genes encode a chlorite-disproportioning enzyme. In several Gram-positive bacteria, Cld-like proteins seem to carry out an important function during heme biosynthesis (which was the reason for renaming the respective genes to *hemQ*), although their exact molecular role is not yet understood (Dailey et al., 2010; Mayfield et al., 2013; Hofbauer et al., 2015). The *in vivo* substrates and natural functions of most Cld-like proteins have not been identified yet.

### Alternative Pathways for Perchlorate Reduction

Studies on the archaeon *A. fulgidus* demonstrated that chlorite dismutase does not necessarily have to be present in a microorganism for complete reduction of chlorine oxyanions. The presence and regeneration of reduced sulfur compounds enables an alternative perchlorate reduction pathway in this archaeon, where chlorite is eliminated by sulfide, forming sulfur compounds of higher oxidation states (S_x_O_y_^z-^) (Liebensteiner et al., 2013, 2014) (Figure 6B). The initial step of perchlorate reduction in *A. fulgidus* (from perchlorate to chlorite) is putatively mediated by a DMSO II oxidoreductase (Af_0174-0176) (Liebensteiner et al., 2013) that is only moderately related to known Pcr. The enzyme has been associated with Nar-type enzymes, but unlike the bacterial Nar, Af_0174-0176 has its catalytic subunit located in the periplasm (Dridge et al., 2006). Such periplasmic Nar-type nitrate reductases were biochemically characterized from three different archaeal species (Yoshimatsu et al., 2000; Afshar et al., 2001; Lledo et al., 2004). Similar to respiratory nitrate reductases of bacteria, the pNar enzymes from *H. meditteranei* and *Pyrobaculum aerophilum* utilize chlorate (Afshar et al., 2001; Martinez-Espinosa et al., 2007). A study on the pNar of *H. meditteranei*, a perchlorate-reducing microorganism (Oren et al., 2014), also demonstrated the enzyme’s ability to reduce perchlorate (Martínez-Espinosa et al., 2015). Interestingly, the thermophilic archaeon *Aeropyrum pernix* previously thought to be an obligate aerobe is able to grow anaerobically with perchlorate as electron acceptor, employing a similar mechanism as *A. fulgidus*. In this case, thiosulfate present in the medium was shown to be responsible for chlorite elimination (Liebensteiner et al., 2015). This sulfur compound-mediated perchlorate reduction occurs more common, especially at high salinity (Barnum et al., 2018).

The periplasmic location of chlorate- and perchlorate-reducing enzymes may be particularly important for complete reduction of chlorine oxyanions in the absence of a Cld, exposing chlorite to potential chemical scavengers, like reduced sulfur compounds. Besides complete perchlorate reduction in *A. fulgidus*, involving an intraspecies “sulfur loop” (Figure 6B), the same task could be fulfilled by interspecies cooperation. Syntrophic communities comprising sulfur-/sulfate-reducing microorganisms and microorganisms that harbor a periplasmic chlorate- or perchlorate-reducing enzyme may enable the reduction of chlorine oxyanions mediated by an interspecies “sulfur loop” (Figure 6C). These putative alternatives of chlorate/perchlorate reduction would extend the diversity and flexibility of the metabolism beyond the canonical pathways (Figure 6A), especially in highly reduced environments.

Enzymes related to Af_0176 of *A. fulgidus* are found in archaea (*Ferroglobus placidus*, Ferp_0124) and bacteria (*Desulfosporosinus meridiei*, Dsmer_2075; *Desulfitobacterium dehalogenans*, Desde_0947; *D. dechloroeliminans*, Desdi_0326; *Carboxydothermus hydrogenoformans*, Cyh_2082; *Moorella thermoacetica*, Moth_1908). For some of these enzymes, the potential function as a pNar was discussed earlier (Martinez-Espinosa et al., 2007). Similar to halobacteria and *A. fulgidus*, these microorganisms may possibly be able to reduce chlorine oxyanions, and hence obtain energy for growth, by using pNar.

It was demonstrated that *Sulfurospirillum multivorans* has the ability to reduce and grow with several sulfur compounds and perchlorate as electron acceptors (Goris et al., 2014). The genome sequence of this well-studied OHRB (Scholz-Muramatsu et al., 1995) indicates an enormous metabolic flexibility. However, the bacterium can probably not rely on chlorite dismutase for complete perchlorate reduction, since no *cld*/*cld*-like genes were found in the genome (Goris et al., 2014). Whether *S. multivorans* employs similar strategies as *A. fulgidus* to eliminate chlorite is yet to be investigated.

### Substrate Range and Accelerated Degradation of Recalcitrant Compounds

Perchlorate and chlorate reducers are able to use a wide range of organic and inorganic electron donors for growth (Coates et al., 1999b; Son et al., 2006; Ju et al., 2008; Nikel et al., 2014; Liebensteiner et al., 2015). The ability of perchlorate and chlorate reducers to produce oxygen by chlorite dismutase activity allows growth on compounds that may require oxygenase-dependent pathways for degradation and are thus difficult to degrade anaerobically. Aromatic and aliphatic hydrocarbons are examples of such compounds. Oxygen production during perchlorate/chlorate reduction may enable these bacteria to employ oxygenase-dependent pathways under seemingly anoxic conditions. This concept offers interesting perspectives for bioremediation (Coates et al., 1998, 1999a; Langenhoff et al., 2009; Hofbauer et al., 2014; Brundrett et al., 2015). We investigated bacteria that are able to grow anaerobically with benzene or alkanes as electron donors and chlorate as electron acceptor. A strain of *Alicycliphilus denitrificans* was isolated that can grow with benzene and some other aromatic hydrocarbons as electron donor and chlorate or oxygen as electron acceptor (Weelink et al., 2007, 2008). This bacterium cannot grow with such compounds when nitrate is the electron acceptor, whereas it can grow with acetate and nitrate. Genome analysis indicated that the bacterium uses oxygenase-dependent pathways for growth with benzene and some other aromatic hydrocarbons (Oosterkamp et al., 2013). This indicates that oxygen formed by dismutation of chlorite is used as terminal electron acceptor and in oxygenase-dependent reactions. Interestingly, in this bacterium all genes needed for chlorate reduction are located on a plasmid (Oosterkamp et al., 2013). Similarly, *Pseudomonas chloritidismutans*, a bacterium that had been isolated with acetate and chlorate, was able to grow with decane and other medium-chain alkanes and chlorate (Mehboob et al., 2009). Further genomic and proteomic analysis provided evidence for the occurrence of intracellular oxygen transfer (Mehboob et al., 2015). In theory, it might also be possible that growth with hydrocarbons and chlorate is a process that occurs by syntrophic cooperation between a bacterium that degrades hydrocarbons with oxygen that is provided by a chlorate-reducing bacterium. Indications for such possibility were obtained from a mixed benzene-chlorate degrading community, but attempts to isolate the respective bacteria were not successful. Thus, unequivocal proof is still lacking (Weelink et al., 2007). However, a coculture of two *Alicycliphilus denitrificans* strains, one of which can use cyclohexanol whereas the second strain can reduce chlorate (Oosterkamp et al., 2013), showed some cyclohexanol degradation with chlorate provided that acetate was added as additional electron donor (Veuskens et al., unpublished data), suggesting the occurrence of interspecies oxygen transfer, albeit a slow process. Chlorite addition to enrichments in which the perchlorate reducer *Dechloromonas agitata* and aerobic methanotrophs (*Methylococcus capsulatus* and *Methylomicrobium album*) were present, enhanced methane oxidation, however, methane oxidation could not be clearly shown with chlorate or perchlorate. Methane oxidation with perchlorate was shown in mixed microbial communities, but who is doing what in these communities is still not clear (Luo et al., 2015; Xie et al., 2018).

## Co-Occurrence and Potential Interconnections Between Chlorination and Dechlorination

The broad range of microbial chlorination and dechlorination mechanisms known to date leads to the hypothesis that the formation and degradation of organochlorines might be linked whereby dechlorinating microorganisms would depend on chlorinating microorganisms for their supply of carbon and energy or terminal electron acceptors. On the other hand, the bulk of chlorine on Earth is present in an inorganic form (Graedel and Keene, 1996), which diminishes the extent to which microbial interconnections in chlorine biochemistry are to be expected at a global scale. For example, the most common microbiologically produced organochlorine, chloromethane, is largely degraded in the troposphere due to oxidation by OH radicals and in the stratosphere by OH oxidation or photochemically (Harper, 2000; Yoshida et al., 2004). However, in contrast to the relatively slow abiotic conversions in the global chlorine cycle, local microbial chlorine cycles may occur if the escape of organochlorines from the biological producer to the atmosphere is limited.

Chlorine cycling at the smallest confined space is the chlorination and dechlorination of differentiation-inducing factor 1 (DIF-1) within cells of the social amoeba *Dictyostelium discoideum*. DIF-1 is involved in the development of the multicellular stage in the amoeba’s life cycle. To tightly regulate the amount of chlorinated DIF-1, the amoeba possesses a refined system that includes both halogenation and dehalogenation reactions, where the chlorination induces dechlorination (Insall et al., 1992; Velazquez et al., 2011). Similarly co-occurring chlorination and dechlorination may happen in other habitats where halogenating and dehalogenating organisms are in close contact. For example, the marine sponge *Aplysina aerophoba* is known to produce a variety of non-volatile brominated and chlorinated secondary metabolites (Turon et al., 2000). The sponge holobiont harbors a large number of bacteria with a variety of FADH_2_-dependent halogenases (Bayer et al., 2013), while the OHRB *Desulfoluna spongiiphila* was isolated from the same sponge species (Ahn et al., 2009). Despite the fact that oxidative mechanisms predominate halogenation (Vaillancourt et al., 2006) and that known OHRB are mostly strict anaerobes (Atashgahi et al., 2016), oxygen might not be a major divide between halogenation and dehalogenation processes. For instance, aerobic microbes that were once thought to rarely perform reductive dechlorination were shown to comprise catabolic reductive dehalogenases (Chen et al., 2013; Payne et al., 2015). Moreover, even the once notoriously strict anaerobic *D. mccartyi* was shown to be resistant and resilient to oxygen exposure in their natural sediment habitats (Atashgahi et al., 2017a). Presence of haloacid dehalogenases was reported in a bacterial isolate hosted by the marine sponge *Hymeniacidon perlevis* (Zhang et al., 2013). Although the halogenation potential of this sponge species remains to be demonstrated, the presence of halogenated secondary metabolites in marine sponges is a rule rather than an exception (Kochanowska-Karamyan and Hamann, 2010).

Adding to this knowledge, there is increasing evidence of co-occurrence of halogenating and dehalogenating microbes and genes in different habitats that have a more pronounced impact as global chlorine source or sink. For example, chloromethane and chloroform are produced as bulk organochlorines by fungi, terrestrial and marine plants, bacteria and unicellular marine eukaryotes (Scarratt and Moore, 1998; Gribble, 2004). Although chloromethane is largely chemically degraded in the troposphere (Harper, 2000; Yoshida et al., 2004), microbial sinks can fix an estimated range of 0.6–1.5 Tg/year of chloromethane before it escapes to the atmosphere (Keppler et al., 2005). Recent metagenomic analyses have shown the presence of both halogenase- and dehalogenase-encoding genes in Arctic tundra and forest soils (Weigold et al., 2016; Zlamal et al., 2017). Moreover, halogenation (Weigold et al., 2016) and anaerobic dehalogenation (Zlamal et al., 2017) were experimentally verified in soil microcosms derived from the same sites.

Chloromethane-degrading bacteria are ubiquitous in soil (Jaeger et al., 2018), and chloromethane-degrading bacteria were isolated from the phyllosphere of plants where the chlorine compounds are produced (Nadalig et al., 2011). A remarkable demonstration of the dependency of dechlorinating bacteria on the production of chlorine compounds is the recent finding that the abundance of chloromethane-degrading bacteria and the *cmuA* gene encoding methyltransferase that catalyzes the initial reaction of methylhalide degradation were much lower for *Arabidopsis thaliana* lacking the gene encoding for *HOL1*, the methyltransferase responsible for most of the chloromethane emissions by the plant (Haque et al., 2017). Furthermore, the *cmuA* gene has also been found to be widely distributed in the oceans (Cox et al., 2012). Similar dependencies of chlorination and dechlorination in marine environments may be expected and awaits experimental validation.

Other examples of co-occurring chlorination and dechlorination reactions is the metabolism of tetrachloro-4-methoxyphenol to chlorophenol by the OHRB *Desulfitobacterium hafniense* strain PCP-1 (Milliken et al., 2004). Tetrachloro-4-methoxyphenol is an environmentally important fungal product due to its potential physiological role during lignin degradation and was detected in composite forest litter (De Jong and Field, 1997). Another coordinated action of halogenating and dehalogenating enzymes has recently been shown to be involved in bacterial biosynthesis of halogenated secondary metabolites. As such, dehalogenation of aromatic organohalogens by a thioredoxin-like dehalogenase was necessary for the production of the highly brominated marine bacterial natural product, pentabromopseudilin (El Gamal et al., 2016).

## Conclusion and Outlook

Despite an already impressive and still increasing number of over 5000 naturally produced organochlorines, a limited set of mechanisms is known that can add or remove chlorine from organic compounds. Whereas chlorination reactions have been a subject of interest for production of natural bioactive compounds, the quest for dechlorination reactions has been largely fuelled by the large-scale contaminations with toxic chlorine compounds. Chlorination and dechlorination reactions use different cofactors, and connections between the formation and degradation of organochlorines might be tenuous. However, examples have been discovered showing dependency of the dechlorinating microorganisms on chlorinating microorganisms for their supply of carbon and energy or terminal electron acceptors. Accordingly, the natural biogeochemical logic dictates a balance between production and mineralization of chlorine compounds to prevent their accumulation in nature. Hence, it remains to be established to what extent naturally occurring chlorine compounds metabolically link halogenating and dehalogenating microbes. Building upon the wealth of current knowledge on specific elements of the chlorine cycle, comprehensive studies are needed to obtain fine-scale understanding of community functions and their interactions within the context of the chlorine cycle, because, except for exemplary evidence, at this point the hypothesis that the microbial formation and degradation of organochlorines are intertwined at significant scales can be neither confirmed nor rejected. To this end, chlorination and dechlorination of natural organic matter should be studied simultaneously and within their natural ecosystems like forest soils. Comparative (meta)genomics should be coupled with downstream functional genomics based on (meta)transcriptomes and (meta)proteomes, biochemical assays and genetic modification studies to generate holistic insights into community dynamics and function, and potential interactions between chlorinators and dechlorinators.

## Author Contributions

HS, AS, and DS planned the initial scope of the review. SA, ML, DJ, and DS prepared the figures. All authors made a substantial, direct and intellectual contribution to the writing, editing, and revision of the manuscript, and approved it for publication.

## Conflict of Interest Statement

The authors declare that the research was conducted in the absence of any commercial or financial relationships that could be construed as a potential conflict of interest.
